# Ponatinib Induces a Procoagulant Phenotype in Human Coronary Endothelial Cells via Inducing Apoptosis

**DOI:** 10.3390/pharmaceutics16040559

**Published:** 2024-04-19

**Authors:** Bálint Krajcsir, Marianna Pócsi, Zsolt Fejes, Béla Nagy, János Kappelmayer, Ildikó Beke Debreceni

**Affiliations:** 1 Department of Laboratory Medicine, Faculty of Medicine, University of Debrecen, H-4032 Debrecen, Hungary; krajcsir.balint@med.unideb.hu (B.K.); pocsi.marianna@med.unideb.hu (M.P.); fejes.zsolt@med.unideb.hu (Z.F.); kappelmayer@med.unideb.hu (J.K.); 2 Laki Kálmán Doctoral School, Faculty of Medicine, University of Debrecen, H-4032 Debrecen, Hungary

**Keywords:** ponatinib therapy, endothelial cell, apoptosis, phosphatidylserine, endothelial microvesicles, thrombin generation

## Abstract

BCR-ABL tyrosine kinase inhibitors (TKIs) are effective drugs in the treatment of patients with chronic myeloid leukemia. However, based on clinical studies, ponatinib was associated with the development of thrombotic complications. Since endothelial cells (ECs) regulate blood coagulation, their abnormal phenotype may play a role in the development of thrombotic events. We here aimed to investigate the effect of ponatinib on the procoagulant activity of cultured endothelial cells in vitro. Human coronary artery endothelial cells (HCAECs) were incubated with 50, 150, and 1000 nM of ponatinib. Subsequently, phosphatidylserine (PS) exposure and endothelial microvesicles (EMVs) were measured by flow cytometry. In addition, EC- and EMV-dependent thrombin generation was analyzed. To investigate pro-apoptotic effects of ponatinib, the level of Bax and Bcl-xL proteins were studied using Western blot and F3, THBD, and VCAM1 mRNAs were quantified by qPCR. Therapeutic concentrations of ponatinib significantly increased PS expression on ECs and the amount of EMVs which significantly shortened the time parameters of thrombin generation. In addition, these changes were associated with an increased ratio of Bax and Bcl-xL proteins in the presence of the decreased THBD mRNA level. Overall, ponatinib enhances the procoagulant activity of ECs via inducing apoptosis, which may contribute to thrombotic events.

## 1. Introduction

Chronic myeloid leukemia (CML) is a hematological malignancy characterized by the uncontrolled and increased growth of myeloid cells which is a consequence of the presence of the Philadelphia chromosome (Ph). This translocation leads to the production of the BCR-ABL1 oncoprotein that enhances the differentiation and proliferation of malignant myeloid cells via its tyrosine kinase activity. The BCR-ABL tyrosine kinase inhibitors (TKIs) are a component of anticancer treatment, and their use dramatically improved the overall survival of CML patients. Imatinib, the first generation of TKIs, was approved by the United States Food and Drug Administration in 2001 [1]. Imatinib has proven to be a very effective drug but has not achieved optimal response in patients who have developed resistance or intolerance to this drug. For this reason, the second-generation TKIs dasatinib, nilotinib, and bosutinib were developed [2]; nevertheless, even these drugs were ineffective in CML patients who possessed the T315I gatekeeper mutation. This genetic alteration later led to the development of the third-generation ponatinib [3]. Its activity is particularly important, since no other drug inhibits this mutant protein, which frequently arises during the treatment with other TKIs (imatinib, dasatinib, nilotinib, and bosutinib) approved for CML and/or Ph^+^ acute lymphoblastic leukemia (ALL) treatment [4]. Hence, ponatinib treatment can also be applied in solid tumors, including thyroid, breast, ovary, and lung cancer, as well as neuroblastoma and rhabdoid tumors [5].

Although ponatinib is highly effective for the treatment of patients with CML, it increases the risk of vascular adverse events (VAEs), particularly the risk of arterial thrombosis associated with myocardial infarction, stroke, and peripheral artery disease [6]. In the 5-year follow-up of the phase 2 PACE trial, the cumulative incidence of arterial occlusive events (AOEs) was 25% [7]. In a recent meta-analysis of several clinical trials on CML patients treated with TKIs, ponatinib therapy was associated with an increased incidence rate for cardiovascular or arteriothrombotic adverse events [8]. 

The development of cardiovascular adverse events (CV-AEs) may be due to the effect of ponatinib on both platelets and endothelial cells (ECs). In previous studies, where platelet reactivity was investigated in this aspect, interestingly, ponatinib showed an inhibitory as well as enhancing effect on platelet function. On the one hand, ponatinib attenuated platelet activation, spreading, granule secretion, and aggregation, likely through the inhibition of a broad spectrum of tyrosine kinase signaling pathways in platelets [9,10,11]. In contrast, in mice treated with ponatinib, von Willebrand factor (vWF)-mediated platelet adhesion and microvascular angiopathy contributed to ischemic wall (motion) abnormalities [12]. Furthermore, ponatinib selectively enhanced agonist-induced platelet reactivity at human therapeutic concentration in mice [13]. From these conflicting studies, it seems that ponatinib may alter the function of other cell types, such as ECs, which may contribute to the development of arterial vascular events. An in vitro study demonstrated that ponatinib inhibited the survival of human umbilical vein endothelial cells (HUVECs) [14]. In addition, in a similar experimental setup, ponatinib reduced angiogenesis via blocking the vascular endothelial growth factor receptor (VEGFR) signaling pathway [15] and induced vascular toxicity [16,17]. The results of these studies support that ponatinib also affects EC function and viability. 

It has long been known that the endothelium plays an important role in cardiovascular disorders via regulating vascular tone, inflammation, angiogenesis, and blood coagulation [18,19,20,21]. Under physiological conditions, the endothelium prevents thrombosis through various anticoagulant and antiplatelet mechanisms. During anticoagulant processes, ECs produce several thrombin inhibitors, as well as protein C-activating receptor (EPCR), thrombomodulin (TM), and tissue factor pathway inhibitor (TFPI) [21]. However, in the case of vascular injury, EC activation or apoptosis may occur, when these cells switch from an anticoagulant to a prothrombotic phenotype. As a result, procoagulant molecules, such as tissue factor (TF) and/or phospholipid (PL) may appear on the surface of ECs, endothelial microvesicles (EMVs) are formed and the level of anticoagulant proteins may decrease. These alterations can serve as biomarkers of endothelial dysfunction and contribute to thrombus formation [22].

The aim of our study was to investigate the effect of ponatinib on the procoagulant properties of ECs in vitro using coronary artery endothelial cell (HCAEC) culture at clinically relevant and supratherapeutic drug concentrations. Furthermore, we intended to clarify the mechanism whereby ponatinib exerts its effect on thrombin generation.

## 2. Materials and Methods

### 2.1. Endothelial Cell Culturing and Treatment

For our experiments, HCAEC line, cell culturing medium, trypsin-EDTA, Hank’s balanced salt solution (HBSS) and dimethyl-sulfoxide (DMSO) were obtained from Sigma-Aldrich (St. Louis, MO, USA). Plastic cell culturing flasks and plates were purchased from Greiner Bio-One (Nürtingen, Germany). Ponatinib was ordered from Cayman Chemical (Ann Arbor, MI, USA). HCAECs were cultured in MesoEndo growth medium in 75 cm^2^ flasks at 5% CO_2_ and 80% humidity. When the cells reached 80–90% confluence in the flask, they were plated in 6-well culture plates (10^5^ cells/well), and after 2 days, the cells were treated for up to 48 h with clinically relevant and supratherapeutic concentrations of ponatinib. The final concentrations of ponatinib were 50, 150, and 1000 nM, DMSO (0.2 *v*/*v* %) was used as a negative control, and tumor necrosis factor alpha (TNF-α) (100 ng/mL; Gibco, Waltham, MA, USA) was used as a positive control. After the treatments, the cells were collected from both the plate and the culture medium.

### 2.2. Phosphatidylserine (PS) Exposure and Microvesicle Count Assay Using a Flow Cytometer

Harvested cells were centrifugated (200× *g*, 5 min, 20 °C) and resuspended in phosphate-buffered saline (PBS; 140 mM NaCl, 2.7 mM KCl, 1.47 mM KH_2_PO_4_, 8.1 mM Na_2_HPO_4_, pH 7.4). For the determination of PS positivity, 50 μL of cell suspension was added to 5 μL of ten-fold concentrated annexin binding buffer (Beckton Dickinson, San Jose, CA, USA) and 5 μL of annexin V FITC (Beckton Dickinson, San Jose, CA, USA). Subsequently, samples were incubated for 15 min at room temperature (RT) in the dark, and then 500 μL of annexin V binding buffer was added. The ratio of annexin V binding cells was determined using a FC500 (Beckman Coulter, Brea, CA, USA) flow cytometer. 

After the separation of cells from the cell medium, the supernatant was further centrifuged (16,000× *g*; 30 min; 20 °C) to obtain endothelial microvesicles (EMVs). After centrifugation, EMVs were washed with PBS and centrifuged again at 16,000× *g* for 30 min. Then, the supernatant was again aspirated and the sediment containing the EMVs was resuspended in a total of 100 μL of annexin binding buffer. For the EMV counting assay, 3 μL of annexin binding buffer (ten-fold concentrated) and 5 μL of annexin V FITC were added to 20 μL of EMV suspension and were incubated in the dark at RT for 15 min and finally 250 μL of annexin V binding buffer was added to the tubes. Size-calibrated beads (Life Technologies, Carlsbad, CA, USA) were used for the size-based identification of EMVs. For this purpose, 0.5, 1, 2, 4, and 6 µm diameter of beads were used. The annexin V positive EMVs were determined using a Canto II flow cytometer (Beckton Dickinson, San Jose, CA, USA) with MED flow rate for 1 min.

### 2.3. Measurement of Thrombin Generating Potential of Endothelial Cells and Microvesicles

Thrombin generation was measured in the presence of ponatinib-treated endothelial cells and cell-derived EMV in the presence of control plasma (PPP) using a Fluoroskan Ascent fluorimeter with Trombinoscope reagents and Thrombinoscope 5.0 software (Trombinoscope BV, Maastricht, The Netherlands). For the preparation of control PPP, control subjects were recruited with the permission of the Ethics Committee of University of Debrecen (RKEB/IKEB 5906-2021). PPP was obtained by the centrifugation of citrated whole-blood sample at 1500× *g* for 15 min at RT, then the PPP was further centrifuged at 10,000× *g* for 10 min at RT. For the detection of thrombin generation, 20 µL of PBS was pipetted into the well of a 96-well black plate and 80 µL of a ponatinib-treated cells + PPP mixture was added in the sample-wells. For the calibrator wells, 80 µL of PPP and 20 µL of calibrator reagent were pipetted. The assembled plate was incubated for 10 min at 37 °C, and the coagulation was initiated by the addition of FluCa substrate (buffer containing peptide-aminomethylcoumarin and calcium). The change of fluorescence signal was measured for 60 min. At the end of the measurement, the Thrombinoscope software drew the thrombogram, which shows the amount of generated thrombin. The thrombogram is characterized by time parameters such as lagtime and time to peak, and quantitative parameters such as thrombin peak and endogenous thrombin potential (ETP). Lagtime is the moment at which the thrombin formation starts. The time to peak shows the time that is required for reaching the thrombin peak. The peak value of thrombin is the highest concentration of formed thrombin, and the ETP shows the total amount of generated thrombin.

For the measurement of thrombin-generating potential of EMVs, 20 µL of EMV suspension was added to 80 µL of PPP and the coagulation was initiated by the addition of FluCa substrate.

### 2.4. Western Blot Analysis of Bax and Bcl-xL Proteins

The quantity of Bax and Bcl-xL proteins in ponatinib-treated cells were analyzed by Western blot. After the treatment, cells were washed in PBS and centrifuged at 200× *g* for 5 min at 20 °C. Subsequently, cells were lysed (1% Triton X-100 in PBS supplemented with protease- and tyrosine phosphatase inhibitor) and sonicated. Cell-lysate samples were incubated in a buffer (62.5 mM Tris, 2% SDS, 10% glycerol, 5% MEA, pH 6.8) for 5 min at 95 °C. The separation of proteins was performed by SDS-polyacrylamide gel (15%). After Western blotting, the membrane was stained by anti-human Bax, Bcl-xL (Cell Signaling Technology, Danvers, MA, USA) or anti-actin (Abcam, Cambridge, UK). After that, biotinylated anti-rabbit IgG (for Bax and Bcl-xL; Invitrogen, Waltham, MA, USA) or anti-mouse IgG-HRP (for actin; Abcam, Cambridge, UK) secondary antibodies were used. Avidin–biotin complex (ABC) was used for the detection of biotinylated antibody. Protein bands were visualized by enhanced chemiluminescence (ECL, Merck Millipore, Burlington, MA, USA).

### 2.5. Investigation of F3, THBD, and VCAM1 mRNA Expression

After treatments, the HCAEC cells were washed with HBSS solution and lysed in 1 mL TRI Reagent (Molecular Research Center, Cincinatti, OH, USA). Total RNA—including messenger RNAs (mRNA)—was extracted from HCAECs following the manufacturer’s protocol. The concentration and purity of separated RNA samples were verified by a NanoDrop 2000 spectrophotometer (Thermo Scientific, Wilmington, DE, USA) and RNA samples were stored at −80 °C before mRNA expression analyses. The real-time quantification of selected mRNAs (F3, THBD and VCAM1) was performed based on the method reported in our previous study [23]. Briefly, complementary DNA (cDNA) was first synthesized using the High-Capacity cDNA Reverse Transcription Kit (Applied Biosystems, Vilnius, Lithuania) according to the manufacturer’s instructions. The initial amount of RNA was 500 ng per reaction. Then, real time-quantitative PCR (RT-qPCR) was performed using LightCycler 480 SYBR Green I Master mix (Roche Diagnostics, Mannheim, Germany) with gene-specific primers (10 µM, Integrated DNA Technologies, Leuven, Belgium). The reactions (95 °C for 10 min, followed by 40 cycles of 95 °C for 10 s and 60 °C for 1 min) were run in triplicates on a QuantStudio 12K Flex qPCR instrument (Applied Biosystems by Life Technologies, Carlsbad, CA, USA). We used the refence gene RPLP0 (36B4) for normalization, and the sequences of these oligonucleotides are listed in Table 1. 

### 2.6. Statistical Analysis

For the statistical analysis, GraphPad Prism 9.1.2 was used. The distribution of the data was determined by the Kolmogorov–Smirnov test. To determine the level of significance of differences between the DMSO- and ponatinib-treated groups, the one-way ANOVA test was used for Gaussian distribution with Tukey’s multiple comparisons test and a Kruskal–Wallis test for non-Gaussian distribution with Dunn’s post hoc test. The comparison between DMSO- and TNF-α-treated samples was performed by unpaired *t*-test or the Mann–Whitney test. Differences were considered significant if the *p*-value was below 0.05. 

## 3. Results

### 3.1. Ponatinib Enhances Phosphatidylserine (PS) Exposure on ECs

**Figure 1:**
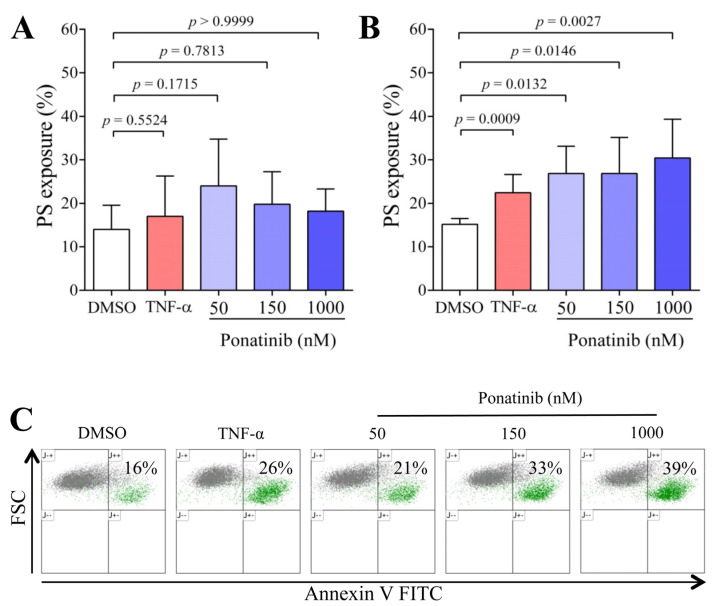
Ponatinib enhances the phosphatidylserine (PS) exposure on endothelial cells (ECs). ECs were incubated with different concentrations of ponatinib (50, 150, and 1000 nM), TNF-α (100 ng/mL) as positive control and vehicle (DMSO) for 24 h (**A**) or 48 h (**B**) at 37 °C. The exposure of PS was determined by FITC-labeled annexin V. The graphs display the percentages of ECs binding FITC-annexin V at 24 h (**A**) and 48 h (**B**). A significant effect was not observed after 24 h, but in the 48 h samples, TNF-α and ponatinib at all applied concentrations significantly enhanced the PS exposure on the surface of ECs. The results are the mean ± SD of 5 experiments/24 h and 7 experiments/48 h. The unpaired *t*-test and Kruskal–Wallis test with Dunn’s correction was used to compare the data of the groups. Representative flow cytometric dot-plots (**C**) show cells with higher annexin V intensity (in green) had a lower FSC value, corresponding to a smaller cell size. Abbreviations: DMSO, dimethyl sulfoxide; FITC, fluorescein isothiocyanate; FSC, forward scatter; PS, phosphatidylserine; TNF-α, tumor necrosis factor alpha.

Upon coagulation, enhanced PS exposure potentiates thrombin generation by harboring coagulation factors. Therefore, we first examined the effect of ponatinib on the expression of PS on ECs by flow cytometry. No significant effect was found either with TNF-α or different concentrations of ponatinib during 24 h treatment (Figure 1A). In contrast, after 48 h, TNF-α (*p* = 0.0009) and all applied concentrations (50, 150 and 1000 nM) of ponatinib significantly enhanced PS exposure (*p* = 0.0132; *p* = 0.0146 and *p* = 0.0027, respectively) on the surface of ECs (Figure 1B). In addition, we also observed that ECs with a higher annexin V intensity (PS positivity) showed lower forward scatter (FSC) values (42 ± 3 vs. 127 ± 12; mean ± SD) than those characterized by low annexin V intensity. This observation implies that ECs with high annexin V positivity have a relatively smaller cell size (Figure 1C), which can be observed in apoptosis.

### 3.2. Ponatinib Reduces the Time Required for the Generation of Thrombin

**Figure 2:**
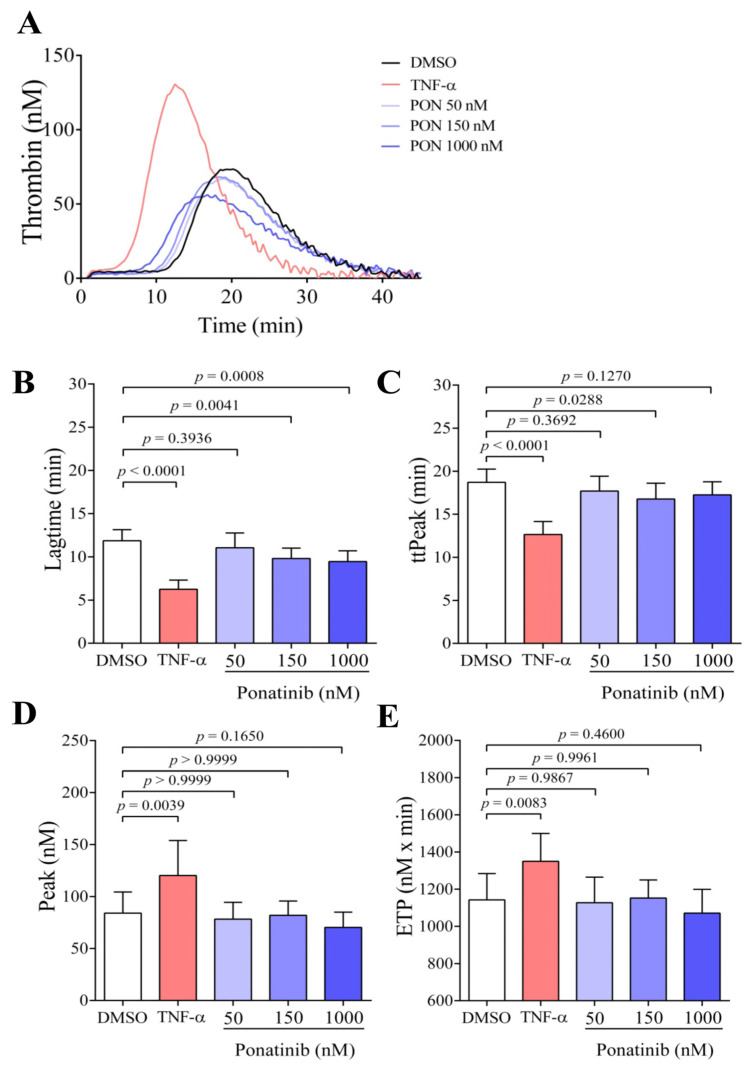
Ponatinib reduces the time required for the generation of thrombin. On panel (**A**), representative thrombin generation curves of ECs are shown at 48 h after the treatment with ponatinib (50, 150, and 1000 nM), TNF-α (100 ng/mL), and DMSO. Lagtime (**B**), time to peak (ttPeak) (**C**), peak thrombin (**D**), and endogenous thrombin potential (ETP) (**E**) parameters were evaluated. Ponatinib significantly reduced the time parameters, lagtime, and ttpeak already at clinically relevant concentrations (150 nM), but did not influence the ETP and peak thrombin. Results are shown as a mean ± SD of 8–11 samples/group. Unpaired *t*-test; the Mann–Whitney test; ordinary one-way ANOVA with Tukey’s correction; or Kruskal–Wallis test with Dunn’s correction was used. Abbreviations: DMSO, dimethyl sulfoxide; PON, ponatinib; TNF-α, tumor necrosis factor alpha.

Based on the result of the PS exposure examination, we wondered whether ponatinib-treated ECs might enhance thrombin generation. After 48 h treatment, ECs were harvested and their thrombin generation potential was measured in the presence of control plasma without the addition of exogenous TF and PL. In this experimental setup, ECs served as a stimuli for the initiation of the coagulation cascade in thrombin generation test (TGT). Figure 2A shows representative thrombin generation curves, which demonstrate that thrombin generation was initiated earlier at 150 and 1000 nM of ponatinib, but the thrombin peak remained unchanged compared to DMSO-treated ECs, whereas after TNF-α treatment, thrombin was formed not just earlier but also with a higher peak. The thrombin generation curve was further characterized by time parameters, such as lagtime and time to peak, and quantitative parameters such as thrombin peak and ETP. After the statistical analysis of the parameters of thrombin generation, the lagtime was significantly reduced at 150 and 1000 nM of ponatinib (Figure 2B, *p* = 0.0041 and *p* = 0.0008, respectively) and time to peak (ttPeak) was also reduced at 150 nM (Figure 2C, *p* = 0.0288). This means that thrombin generation commenced earlier and reached the peak-value earlier in these samples. However, neither the peak-value (Figure 2D) nor ETP (Figure 2E) showed significant changes in response to the ponatinib treatment. Mechanistically, thrombin was generated earlier, but the level of generated thrombin was not increased by ponatinib treatment. Meanwhile, the TNF-α significantly reduced the lagtime (*p* < 0.0001) and ttPeak (*p* < 0.0001), and significantly increased the peak thrombin (*p* = 0.0039) and ETP (*p* = 0.0083).

### 3.3. Ponatinib Treatment Induces Apoptosis of Endothelial Cells

**Figure 3:**
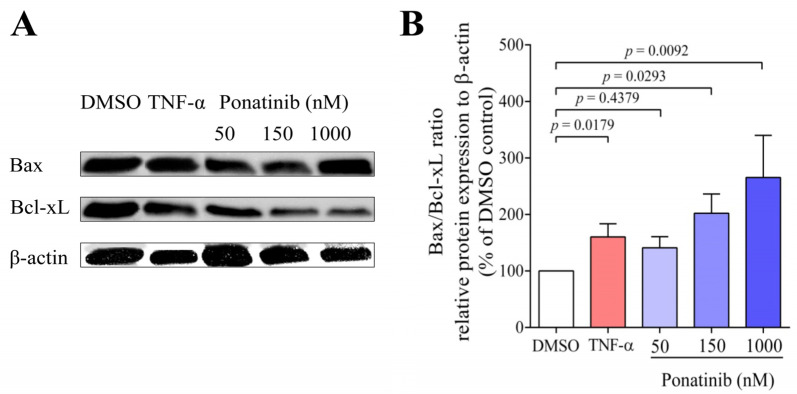
The ponatinib treatment increases the ratio of Bax and Bcl-xL proteins. The quantity of Bax and Bcl-xL proteins was examined by Western blot technique. ECs were exposed to ponatinib and TNF-α (100 ng/mL) for 48 h. On panel (**A**), the original protein bands are shown without the normalization to β-actin. After the normalization of Bax and Bcl-xL bands to β-actin bands, the data showed that ponatinib significantly increased the ratio of Bax and Bcl-xL proteins already at 150 nM (**B**). Data are expressed as the mean ± SEM of five independent experiments. The Mann–Whitney test and Kruskal–Wallis test with Dunn’s correction was used to compare the data of groups. Abbreviations: DMSO, dimethyl sulfoxide; TNF-α, tumor necrosis factor alpha.

Since increased PS exposure may occur during EC activation or apoptosis, the quantity of pro-apoptotic Bax and anti-apoptotic Bcl-xL proteins was examined after 48 h of ponatinib and TNF-α treatment. We found that the ponatinib concentration-dependently increased the ratio of Bax/Bcl-xL protein (Figure 3A) and similar changes were observed after TNF-α treatment. Based on the result of statistical analysis (Figure 3B), ponatinib significantly increased the ratio of Bax and Bcl-xL proteins already at 150 nM (*p* = 0.0293) and the difference was more pronounced at 1000 nM (*p* = 0.0092). Similarly, TNF-α also significantly increased the ratio of Bax and Bcl-xL proteins (*p* = 0.0179).

### 3.4. Ponatinib Enhances the Formation of Endothelial Microvesicles (EMVs)

**Figure 4:**
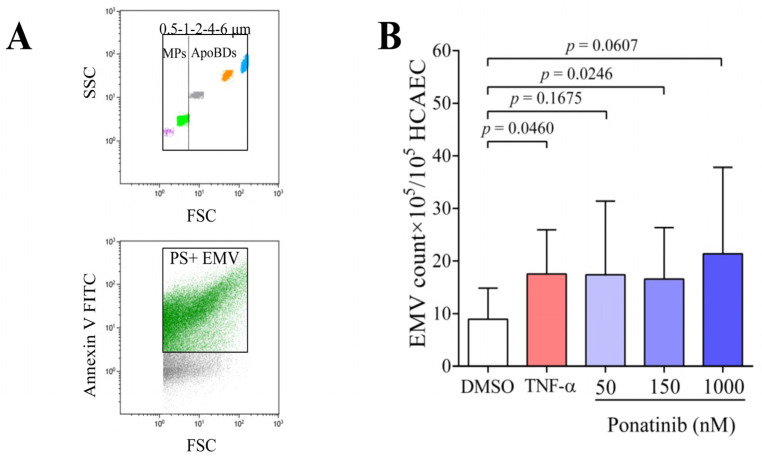
Ponatinib treatment enhances the formation of endothelial microvesicles (EMVs). EMVs were examined by flow cytometry from the supernatant of ponatinib- (50, 150, and 1000 nM), TNF-α (100 ng/mL), and DMSO-treated ECs after 48 h of the treatment. Size-calibrated beads (diameter: 0.5, 1, 2, 4, 6 μm) were used to identify the microparticles (MPs) and apoptotic bodies (ApoBDs) (**A**; upper dot-plot); subsequently only the phosphatidylserine (PS) positive (annexin V positive) EMVs (MPs + ApoBDs) were analyzed (**A**; lower dot-plot). Ponatinib significantly increased the count of EMVs already at 150 nM (**B**). The results are represented as mean ± SD of 7 different experiments. Unpaired *t*-test and ordinary one-way ANOVA with Tukey’s correction was used to compare the data of groups. Abbreviations: DMSO, dimethyl sulfoxide; FITC, fluorescein isothiocyanate; HCAEC, human coronary artery endothelial cell; EMV, endothelial microvesicle; FSC, forward scatter; SSC, side scatter; TNF-α, tumor necrosis factor alpha.

During apoptosis, from apoptotic cells, two types of microvesicles (MV) are generated: microparticles (MPs; 0.1–1 μm) and apoptotic bodies (ApoBDs; 1–6 μm). MVs are PS-positive and may carry TF, depending on the type of activated or apoptotic cell and the mechanism of MV generation. For the identification of EMVs, we used size-calibrated beads to identify boundaries of microparticles (MPs) and apoptotic bodies (ApoBDs) on flow cytometric dot-plot (Figure 4A, upper dot-plot). Subsequently, only PS-positive EMVs (MPs + ApoBDs) were analyzed after annexin V labeling (Figure 4A, lower dot-plot), as these are the EMVs that promote coagulation. The amount of PS-positive EMVs was significantly increased after ponatinib treatment (Figure 4B) already at 150 nM (*p* = 0.0246), and a similar increment was observed after TNF-α treatment (*p* = 0.0460). TNF-α treatment increased the number of EMVs by an average of 97%, and similarly, ponatinib treatment by 95, 86, and 113% (50, 150, and 1000 nM, respectively) compared to the DMSO control.

### 3.5. Ponatinib-Generated Endothelial Microvesicles (EMVs) Reduce the Time Parameters of Thrombin Generation

**Figure 5:**
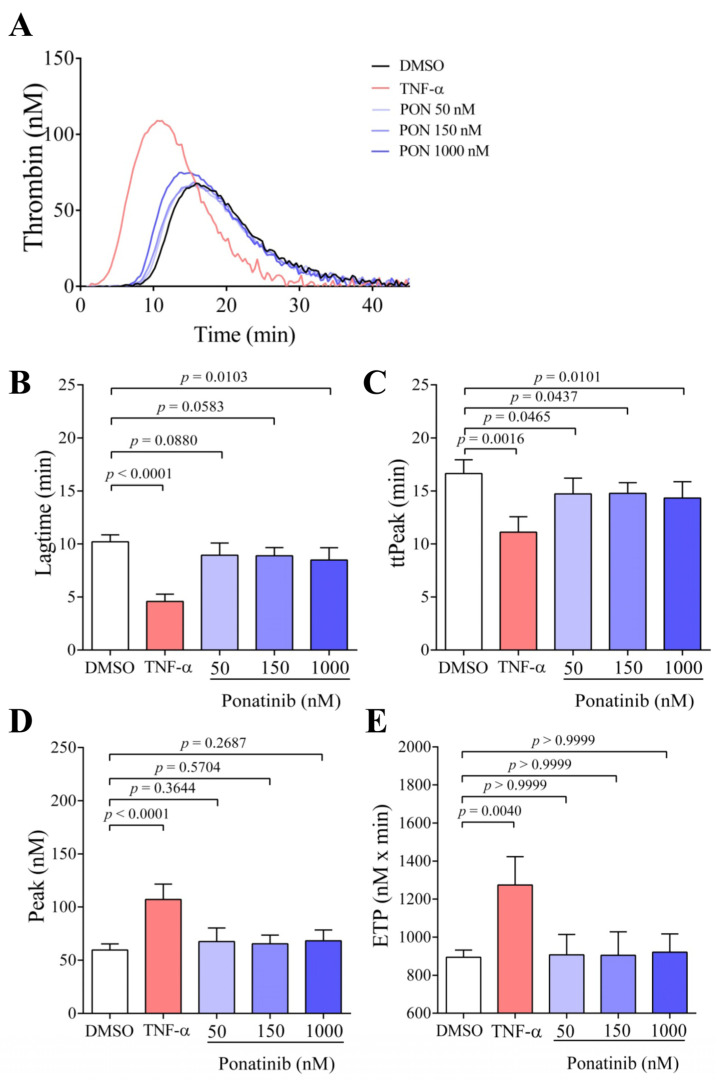
Ponatinib-generated EMVs reduce the time required for the generation of thrombin. After 48 h treatment, the thrombin-generating potential of separated EMVs was examined. Representative thrombin generation curves are shown after ponatinib (50, 150 and 1000 nM), TNF-α (100 ng/mL), and DMSO treatment (**A**). Lagtime (**B**), time to peak (ttPeak) (**C**), peak thrombin (**D**), and endogenous thrombin potential (ETP) (**E**) parameters were evaluated. EMVs separated from ponatinib-treated sample significantly reduced the time parameters, lagtime and ttpeak already at clinically relevant concentration of ponatinib (150 nM) but did not influence ETP and peak thrombin. The results are shown as the mean ± SD of 5–11 samples/group. Unpaired *t*-test or the Mann–Whitney test and ordinary one-way ANOVA with Tukey’s correction or Kruskal–Wallis test with Dunn’s correction was used to compare the data of groups. Abbreviations: DMSO, dimethyl sulfoxide; PON, ponatinib; TNF-α, tumor necrosis factor alpha.

We examined the effect of isolated EMVs on thrombin generation in the absence of exogenous PL and TF as well as in the presence of control plasma. Using the applied experimental setup, EMVs served as a trigger for thrombin generation, because thrombin formation started earlier in the presence of ponatinib-generated EMVs compared to EMVs generated in the presence of the vehicle DMSO (Figure 5A). Importantly, 150 nM ponatinib-generated EMVs almost significantly reduced the lagtime (Figure 5B, *p* = 0.0583), which became significant at 1000 nM ponatinib produced EMVs (*p* = 0.0103). In addition, ponatinib-generated EMVs at 50, 150, and 1000 nM also significantly reduced ttPeak (Figure 5C, *p* = 0.0465; *p* = 0.0437 and *p* = 0.010, respectively); however, we did not observe any changes in the case of thrombin peak (Figure 5D) and ETP (Figure 5E). However, EMVs induced by TNF-α significantly reduced lagtime (*p* < 0.0001) and ttPeak (*p* = 0.0016) and significantly increased the peak thrombin (*p* < 0.0001) and ETP (*p* = 0.0040). These observations suggest that thrombin generation started earlier and reached the peak value much earlier in the presence of EMVs generated by ponatinib.

### 3.6. Ponatinib Treatment Did Not Increase the Quantity of Tissue Factor but Decrease the Level of Thrombomodulin mRNA in HCAEC

**Figure 6:**
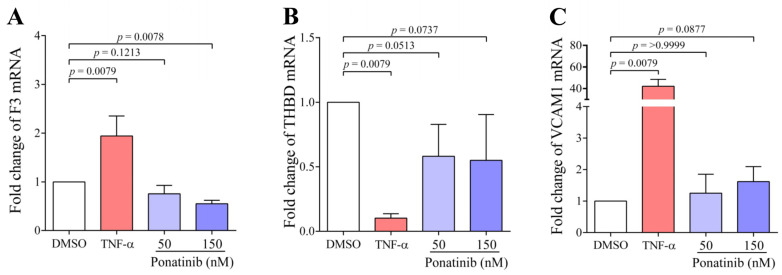
The quantification of the mRNA of the tissue factor (TF), thrombomodulin (TM), and VCAM1 in ECs after ponatinib treatment. HCAECs were treated with clinically relevant concentrations of ponatinib (50, 150 nM), TNF-α (100 ng/mL) and DMSO for 48 h. Subsequently, the procoagulant protein TF (F3) (**A**) and anticoagulant receptor, TM (THBD) (**B**) and VCAM-1 (**C**) were examined at mRNA level. F3 and VCAM1 mRNA levels were significantly increased, while the THBD mRNA level was significantly decreased after TNF-α treatment. However, ponatinib treatment did not increase the TF mRNA level, but considerably decreased the TM and increased VCAM1 mRNA levels. The results are shown as the mean ± SD of 5 samples/group. The Mann–Whitney test and Kruskal–Wallis test with Dunn’s correction was used to compare the data of groups. Abbreviations: DMSO, dimethyl sulfoxide; PON, ponatinib; TNF-α, tumor necrosis factor alpha.

Since the differences in these parameters above were observed at clinically relevant concentrations of ponatinib (50, 150 nM), we further used only these concentrations of ponatinib during 48 h treatment. We wondered whether the expression of anti- and procoagulant proteins was altered during ponatinib or TNF-α treatment in ECs. Hence, the expression of the procoagulant TF and anticoagulant TM proteins at the mRNA level were examined after ponatinib or TNF-α treatment. We found that ponatinib treatment did not enhance F3 mRNA level (Figure 6A) but the level of THBD mRNA was decreased (Figure 6B) and the effect was of borderline significance at 50 (*p* = 0.0513) and 150 nM (*p* = 0.0737). In contrast, the TNF-α treatment significantly increased F3, and downregulated THBD dramatically (*p* = 0.0079, *p* = 0.0079, respectively). During EC activation, not only the level of coagulant proteins, such as TF and TM, but also the level of adhesion proteins may be altered; therefore, we also examined the level of VCAM1 mRNA (Figure 6C). We found in TNF-α-treated samples a dramatically increased VCAM1 mRNA (*p* = 0.0079), while ponatinib treatment slightly increased the mRNA of VCAM1 only at 150 nM (*p* = 0.0877).

## 4. Discussion

In the last decade, several in vitro studies have highlighted the capacity of ponatinib to affect endothelial homeostasis. These studies in part were performed to find out which molecular mechanisms affect the ECs that may underlie the development of cardiovascular or arteriothrombotic adverse events elicited by ponatinib therapy [6]. Ponatinib has been shown to impair EC viability, wound healing, and tube formation [24,25]. 

It has long been known that healthy endothelial cells have anti-inflammatory and antithrombotic effects; however, during EC activation or apoptosis, these properties are shifted in the opposite direction. As a result, ECs display a prothrombotic and proinflammatory phenotype [26].

In our study, we examined for the first time the direct effect of ponatinib on EC procoagulant properties and their hemostatic consequences. For this purpose, the HCAEC line was treated with therapeutic (50, 150 nM) and supratherapeutic (1000 nM) concentrations of ponatinib [3], and TNF-α (100 ng/mL) was used as a positive control. TNF-α acts as a potent activator of ECs, and activated ECs have been associated with vascular EC injury in the development of vasculitis, thrombosis, and other vascular diseases. These processes are mediated by leukocyte adhesion molecules, vasoactive mediators, inflammatory cytokines, chemokines, and procoagulant molecules. If the EC activation process is uncontrolled, this can progress into EC apoptosis [27]. 

Among procoagulant molecules, we first examined PS exposure, which binds coagulation factors and propagates thrombin formation. We found that ponatinib significantly increased the PS exposure on the surface of ECs after the 48 h treatment period, and this was associated with an increased count of PS-positive EMVs already at a 150 nM concentration of ponatinib. Our observation was consistent with the results of the Haguet’s group, when ponatinib increased annexin V binding in a dose-dependent manner on HUVECs, but it was only experienced after 72 h of the treatment [17]. A change in phospholipid asymmetry is associated with PS exposure and this is a fundamental feature of apoptosis. During this process, cultured cells rapidly contract, round up, and are released into the culture medium [26]. We also investigated the cells from the culture medium, which may explain why we observed a significant increase in PS expression already after 48 h. Subsequently, we investigated the effect of ponatinib-treated cells and cell-derived EMVs on thrombin generation assay (TGA) parameters. EMVs arise from ECs during cell activation and apoptosis. These membrane fragments are not only PS-positive but are also able to transport procoagulant molecules, such as TF, which can promote thrombin generation [28]. We found that both ponatinib-treated cells and ponatinib-generated EMVs significantly shortened the time parameters of TGA, which means that thrombin was generated earlier and reached the peak value sooner. During further investigations, the results of our Western blot assay confirmed that the observed changes are the consequence of the EC apoptosis-inducing effect of this drug, as ponatinib increased the ratio of Bax and Bcl-xL proteins. This result was also supported by the findings of other investigators that were observed in a zebrafish model [29]. In our study, the TNF-α treatment increased the PS expression, enhanced the formation of EMVs, resulted in earlier and enhanced thrombin generation, and also increased the ratio of Bax/Bcl-xL. These effects of TNF-α treatment on ECs have been published earlier and confirm the validity of our results [30,31]. In addition, in our mRNA study, we observed that the ponatinib treatment did not increase the level of procoagulant TF (F3) and slightly reduced the level of anticoagulant TM (THBD), which may explain the finding that the amount of thrombin in TGA was not enhanced. In contrast, TNF-α significantly increased the amount of TF and significantly and dramatically reduced the level of TM, which resulted in the increased amount of thrombin in TGA. The TNF-α-induced changes are consistent with earlier published data. In those in vitro studies, a significantly elevated TF expression was found in HUVEC after 18 h TNF-α treatment (10 ng/mL), both at mRNA and protein levels [32]. On the contrary, the TNF-α treatment dramatically decreased the level of TM in HUVEC after 6 h both at mRNA and protein levels [33]. It is known that TNF-α can disturb the hemostatic balance on the surface of ECs by perturbing the expression of several pro- and anticoagulant factors, such as TF [34] and TM [33], and thus can facilitate the thrombin formation that may result in thrombosis. In the early stage of EC activation, the loss of anticoagulant TM was observed, and the subsequent process of EC activation leads to the de novo synthesis of TF [27], while the apoptosis of ECs is associated with increased procoagulant properties through the redistribution of PS. Furthermore, in apoptosis, there is also a loss of anticoagulant surface components, including TM [28]. In our study, we also measured the mRNA level of VCAM1, another protein that is associated with late EC activation. We found that the TNF-α-induced expression of VCAM1 was markedly increased, while ponatinib only slightly increased VCAM1 mRNA at a 150 nM concentration. Our VCAM1 mRNA results are in line with the results of Huang et al., where TNF-α (10 ng/mL) significantly increased the VCAM1 expression at the protein level in HUVECs after 6 h of treatment [35]. Furthermore, our observation that the ponatinib-induced increase in the VCAM1 mRNA level is consistent with the result of an in vitro study at the protein level [36]. In addition, the same changes were observed at mRNA level by Paez-Mayorga et al. [37]; however, they found augmented VCAM1 mRNA already after 24 h of ponatinib treatment in human aortic endothelial cell culture. After 24 h ponatinib treatment, we did not observe a significant elevation in PS exposure, which means that ponatinib did not induce apoptosis; however, cell activation occurred within this short time interval.

Our results confirm the activation and apoptotic effect of ponatinib on HCAECs resulting in increased thrombin generation. This is due to the increased PS expression on the cell surface, the increased EMV formation and the reduced TM level. These changes may contribute to the arterial thrombosis-inducing effect of ponatinib. 

This study has some limitations. In our study, we examined whether a clinically relevant concentration of ponatinib induces the EC apoptosis and procoagulant potential of ECs. In our study, we illustrate ponatinib-induced EC apoptosis and its potential hemostatic consequences; however, we did not examine the ponatinib-induced early EC activation and its effect on thrombin generation. In future in vitro experiments, it would be useful to examine the effect of ponatinib on early EC activation and coagulation.

Furthermore, EC also has a fibrinolytic effect in hemostasis. Therefore, the investigation of the effect of ponatinib on the fibrinolytic properties of ECs can provide a more detailed picture of the development of thrombotic cardiovascular side effects arising from ponatinib treatment.

## 5. Conclusions

The development of ponatinib-associated cardiovascular adverse events (CV-AEs) may be due to the effect of ponatinib on endothelial cells (ECs). In our current study, we describe the ponatinib-induced procoagulant activity of ECs. Ponatinib treatment elevates the exposure of PS on ECs and enhances the formation of EMVs via inducing EC apoptosis. These cellular changes can reduce the time required for thrombin generation, and these prothrombotic effects may contribute to the development of CV-AE.

## Figures and Tables

**Table 1:** Sequences of primers, for the quantitative analysis (Rt-qPCR) of messenger RNAs (mRNAs).

Messenger RNAs	Forward Primers for RT-qPCR	Reverse Primers for RT-qPCR	Amplicon Length
F3	5′-CCCAGAGTTCACACCTTACCT-3′	5′-CGTTCATCTTCTACGGTCACA-3′	102 nt
THBD	5′-GCTCCCCTCGGCTTACAG-3′	5′-GTTCTCCACGCTGCAGTC-3′	102 nt
VCAM1	5′-TGGACATAAGAAACTGGAAAAGG-3′	5′-GATTTCTGGATCTCTAGGGAATGA-3′	67 nt
RPLP0 (36B4)	5′-ATGCAGCAGATCCGCATGT-3′	5′-TCATGGTGTTCTTGCCCATCA-3′	64 nt

## Data Availability

The data presented in this study are available upon request from the corresponding author.

## References

[1] O’Brien S.G., Guilhot F., Larson R.A., Gathmann I., Baccarani M., Cervantes F., Cornelissen J.J., Fischer T., Hochhaus A., Hughes T. (2003). Imatinib compared with interferon and low-dose cytarabine for newly diagnosed chronic-phase chronic myeloid leukemia. N. Engl. J. Med..

[2] Massimino M., Stella S., Tirrò E., Pennisi M.S., Vitale S.R., Puma A., Romano C., Di Gregorio S., Tomarchio C., Di Raimondo F. (2020). ABL1-directed inhibitors for CML: Efficacy, resistance and future perspectives. Anticancer Res..

[3] Cortes J.E., Kantarjian H., Shah N.P., Bixby D., Mauro M.J., Flinn I., O’Hare T., Hu S., Narasimhan N.I., Rivera V.M. (2012). Ponatinib in refractory Philadelphia chromosome-positive leukemias. N. Engl. J. Med..

[4] Soverini S., Colarossi S., Gnani A., Rosti G., Castagnetti F., Poerio A., Iacobucci I., Amabile M., Abruzzese E., Orlandi E. (2006). Contribution of ABL kinase domain mutations to imatinib resistance in different subsets of Philadelphia-positive patients: By the GIMEMA Working Party on Chronic Myeloid Leukemia. Clin. Cancer Res..

[5] Musumeci F., Greco C., Grossi G., Molinari A., Schenone S. (2018). Recent studies on ponatinib in cancers other than chronic myeloid leukemia. Cancers.

[6] Haguet H., Douxfils J., Chatelain C., Graux C., Mullier F., Dogné J.M. (2018). BCR-ABL tyrosine kinase inhibitors: Which mechanism(s) may explain the risk of thrombosis?. TH Open.

[7] Cortes J.E., Kim D.W., Pinilla-Ibarz J., le Coutre P.D., Paquette R., Chuah C., Nicolini F.E., Apperley J.F., Khoury H.J., Talpaz M. (2018). Ponatinib efficacy and safety in Philadelphia chromosome-positive leukemia: Final 5-year results of the phase 2 PACE trial. Blood.

[8] Jain P., Kantarjian H., Boddu P.C., Nogueras-González G.M., Verstovsek S., Garcia-Manero G., Borthakur G., Sasaki K., Kadia T.M., Sam P. (2019). Analysis of cardiovascular and arteriothrombotic adverse events in chronic-phase CML patients after frontline TKIs. Blood Adv..

[9] Neelakantan P., Marin D., Laffan M., Goldman J., Apperley J., Milojkovic D. (2012). Platelet dysfunction associated with ponatinib, a new pan BCR-ABL inhibitor with efficacy for chronic myeloid leukemia resistant to multiple tyrosine kinase inhibitor therapy. Haematologica.

[10] Loren C.P., Aslan J.E., Rigg R.A., Nowak M.S., Healy L.D., Gruber A., Druker B.J., McCarty O.J.T. (2015). The BCR-ABL inhibitor ponatinib inhibits platelet immunoreceptor tyrosine-based activation motif (ITAM) signaling, platelet activation and aggregate formation under shear. Thromb. Res..

[11] Mezei G., Batár P., Kozma L., Illés Á., Kappelmayer J., Debreceni I.B. (2021). Ponatinib exerts an inhibitory effect on collagen-induced platelet aggregation and generation of coated-platelets. Anticancer Res..

[12] Latifi Y., Moccetti F., Wu M., Xie A., Packwood W., Qi Y., Ozawa K., Shentu W., Brown E., Shirai T. (2019). Thrombotic microangiopathy as a cause of cardiovascular toxicity from the BCR-ABL1 tyrosine kinase inhibitor ponatinib. Blood.

[13] Merkulova A., Mitchell S.C., Stavrou E.X., Forbes G.L., Schmaier A.H. (2019). Ponatinib treatment promotes arterial thrombosis and hyperactive platelets. Blood Adv..

[14] Gover-Proaktor A., Granot G., Shapira S., Raz O., Pasvolsky O., Nagler A., Lev D.L., Inbal A., Lubin I., Raanani P. (2017). Ponatinib reduces viability, migration, and functionality of human endothelial cells. Leuk. Lymphoma.

[15] Ai N., Chong C.M., Chen W., Hu Z., Su H., Chen G., Lei Wong Q.W., Ge W. (2018). Ponatinib exerts anti-angiogenic effects in the zebrafish and human umbilical vein endothelial cells via blocking VEGFR signaling pathway. Oncotarget.

[16] Madonna R., Pieragostino D., Cufaro M.C., Doria V., Del Boccio P., Deidda M., Pierdomenico S.D., Dessalvi C.C., De Caterina R., Mercuro G. (2020). Ponatinib-induced vascular toxicity. J. Clin. Med..

[17] Haguet H., Bouvy C., Delvigne A.S., Modaffari E., Wannez A., Sonveaux P., Dogné J.M., Douxfils J. (2020). The risk of arterial thrombosis in patients with chronic myeloid leukemia treated with second and third generation BCR-ABL tyrosine kinase inhibitors may be explained by their impact on endothelial cells: An in-vitro study. Front. Pharmacol..

[18] Galley H.F., Webster N.R. (2004). Physiology of the endothelium. Br. J. Anaesth..

[19] Krüger-Genge A., Blocki A., Franke R.P., Jung F. (2019). Vascular endothelial cell biology: An update. Int. J. Mol. Sci..

[20] Bochenek M.L., Schäfer K. (2019). Role of endothelial cells in acute and chronic thrombosis. Hamostaseologie.

[21] Neubauer K., Zieger B. (2022). Endothelial cells and coagulation. Cell Tissue Res..

[22] Yau J.W., Teoh H., Verma S. (2015). Endothelial cell control of thrombosis. BMC Cardiovasc. Disord..

[23] Fejes Z., Czimmerer Z., Szük T., Póliska S., Horváth A., Balogh E., Jeney V., Váradi J., Fenyvesi F., Balla G. (2018). Endothelial cell activation is attenuated by everolimus via transcriptional and post-transcriptional regulatory mechanisms after drug-eluting coronary stenting. PLoS ONE.

[24] Madonna R., Barachini S., Moscato S., Ippolito C., Mattii L., Lenzi C., Balistreri C.R., Zucchi R., De Caterina R. (2022). Sodium-glucose cotransporter type 2 inhibitors prevent ponatinib-induced endothelial senescence and disfunction: A potential rescue strategy. Vascul. Pharmacol..

[25] Gopal S., Lu Q., Man J.J., Baur W., Rao S.P., Litichevskiy L., Papanastasiou M., Creech A.L., DeRuff K.C., Mullahoo J. (2018). A phosphoproteomic signature in endothelial cells predicts vascular toxicity of tyrosine kinase inhibitors used in CML. Blood Adv..

[26] Bombeli T., Karsan A., Tait J.F., Harlan J.M. (1997). Apoptotic vascular endothelial cells become procoagulant. Blood.

[27] Zhang J., Defelice A.F., Hanig J.P., Colatsky T. (2010). Biomarkers of endothelial cell activation serve as potential surrogate markers for drug-induced vascular injury. Toxicol. Pathol..

[28] Paone S., Baxter A.A., Hulett M.D., Poon I.K.H. (2019). Endothelial cell apoptosis and the role of endothelial cell-derived extracellular vesicles in the progression of atherosclerosis. Cell. Mol. Life Sci..

[29] Lin S., Liu X., Sun A., Liang H., Li Z., Ye S., Ma H., Fan W., Shen C., Jin M. (2023). Qilong capsule alleviates ponatinib-induced ischemic stroke in a zebrafish model by regulating coagulation, inflammation and apoptosis. J. Ethnopharmacol..

[30] Zhou P., Lu S., Luo Y., Wang S., Yang K., Zhai Y., Sun G., Sun X. (2017). Attenuation of TNF-α induced inflammatory injury in endothelial cells by ginsenoside Rb1 via inhibiting NF-κB, JNK and p38 signaling pathways. Front. Pharmacol..

[31] Bacci M., Cancellara A., Ciceri R., Romualdi E., Pessi V., Tumminello F., Fantuzzi M., Donadini M.P., Lodigiani C., Della Bella S. (2023). Development of personalized thrombogenesis and thrombin generation assays to assess endothelial dysfunction in cardiovascular diseases. Biomedicines.

[32] Szotowski B., Antoniak S., Poller W., Schultheiss H.P., Rauch U. (2005). Procoagulant soluble tissue factor is released from endothelial cells in response to inflammatory cytokines. Circ. Res..

[33] Nan B., Lin P., Lumsden A.B., Yao Q., Chen C. (2005). Effects of TNF-α and curcumin on the expression of thrombomodulin and endothelial protein C receptor in human endothelial cells. Thromb. Res..

[34] Cuccuini W., Poitevin S., Poitevin G., Dignat-George F., Cornillet-Lefebvre P., Sabatier F., Nguyen P. (2010). Tissue factor up-regulation in proinflammatory conditions confers thrombin generation capacity to endothelial colony-forming cells without influencing non-coagulant properties in vitro. J. Thromb. Haemost..

[35] Huang W.Y., Wang J., Liu Y.M., Zheng Q.S., Li C.Y. (2014). Inhibitory effect of Malvidin on TNF-α-induced inflammatory response in endothelial cells. Eur. J. Pharmacol..

[36] Wang Y., Travers R.J., Farrell A., Lu Q., Bays J.L., Stepanian A., Chen C., Jaffe I.Z. (2023). Differential vascular endothelial cell toxicity of established and novel BCR-ABL tyrosine kinase inhibitors. PLoS ONE.

[37] Paez-Mayorga J., Chen A.L., Kotla S., Tao Y., Abe R.J., He E.D., Danysh B.P., Hofmann M.C., Le N.T. (2018). Ponatinib activates an inflammatory response in endothelial cells via ERK5 SUMOylation. Front. Cardiovasc. Med..

